# Evidence of enhanced reproductive performance and lack‐of‐fitness costs among soybean aphids, *Aphis glycines*, with varying levels of pyrethroid resistance

**DOI:** 10.1002/ps.6820

**Published:** 2022-03-03

**Authors:** Ivair Valmorbida, Brad S. Coates, Erin W. Hodgson, Molly Ryan, Matthew E. O’Neal

**Affiliations:** ^1^ Department of Entomology Iowa State University Ames IA USA; ^2^ United States Department of Agriculture, Agricultural Research Service, Corn Insects & Crop Genetics Research Ames IA USA; ^3^ Corteva Agriscience, Agriculture Division of DowDuPont Dallas Center IA USA

**Keywords:** lambda‐cyhalothrin, bifenthrin, *vgsc* mutations, insecticide, IRM

## Abstract

**BACKGROUND:**

Foliar application of insecticides is the main strategy to manage soybean aphid, *Aphis glycines* (Hemiptera: Aphididae), in the northcentral United States. Subpopulations of *A. glycines* have multiple nonsynonymous mutations in the voltage‐gated sodium channel (*vgsc*) genes that are associated with pyrethroid resistance. We explored if fitness costs are associated with phenotypes conferred by *vgsc* mutations using life table analyses. We predicted that there would be significant differences between pyrethroid susceptibility and field‐collected, parthenogenetic isofemale clones with differing, nonsynonymous mutations in *vgsc* genes.

**RESULTS:**

Estimated resistance ratios for the pyrethroid‐resistant clones ranged from 3.1 to 37.58 and 5.6 to 53.91 for lambda‐cyhalothrin and bifenthrin, respectively. Although life table analyses revealed some biological and demographic parameters to be significantly different among the clonal lines, there was no association between levels of pyrethroid resistance and a decline in fitness. By contrast, one of the most resistant clonal lines (SBA‐MN1‐2017) had a significantly higher finite rate of increase, intrinsic rate of increase and greater overall fitness compared to the susceptible control and other pyrethroid‐resistant clonal lines.

**CONCLUSIONS:**

Our life history analysis suggests that there are no negative pleotropic effects associated with the pyrethroid resistance in the clonal *A. glycines* lines used in this study. We discuss the potential impact of these results on efficacies of insecticide resistance management (IRM) and integrated pest management (IPM) plans directed at delaying the spread of pyrethroid‐resistant *A. glycines*.

## Graphical abstract

We collected soybean aphids from commercial soybean fields with varying genotypes and phenotypes determined by toxicity bioassays and the presence of nonsynonymous mutations associated with pyrethroid resistance. We used life table analyses to evaluate if fitness costs are associated with pyrethroid‐resistant soybean aphids.

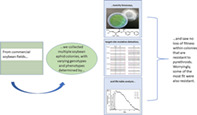

© 2022 The Authors. *Pest Management Science* published by John Wiley & Sons Ltd on behalf of Society of Chemical Industry. This article has been contributed to by U.S. Government employees and their work is in the public domain in the USA.

## 1 INTRODUCTION

The use of pyrethroids is an integral part of global insect pest management strategies, accounting for 15% of the market share worldwide.^1^ Pyrethroids bind to the voltage‐gated sodium channel (vgsc) protein, which alters function of the pore channel, causing repetitive neurological impulses, and resulting in paralysis then death of the insect.^2^, ^3^, ^4^, ^5^ The frequent use and duration of exposure to this insecticide class has contributed to the widespread occurrence of resistance in populations of many insect pests.^1^, ^5^, ^6^ In general, two mechanisms confer resistance to pyrethroids, increased activity or expression of cytochrome P450 monooxygenases (P450), glutathione transferase (GST), or esterase detoxification enzymes, or amino acid substitutions that alter the target site domains of the *vgsc* gene.^2^, ^7^, ^8^, ^9^, ^10^, ^11^

Pyrethroid resistance in several insect species is associated with mutations that alter amino acid sequences in domain II (DII) α‐helical segments 4–6 (S4–6), DIIIS6–DIVS1 and DIVS4–S6 regions of the *vgsc* gene.^2^, ^3^, ^4^, ^9^ Specifically, a knockdown resistance (*kdr*) mutation causing a leucine‐to‐phenylalanine substitution at amino acid position 1014 (L1014F) in DIIS4–S6 of the house fly (*Musca domestica*) vgsc protein is associated with low‐to‐moderate levels of pyrethroid resistance.^4^, ^12^, ^13^ Increased pyrethroid resistance happens when the L1014F mutation co‐occurs with a methionine‐to‐threonine substitution at *vgsc* position 918 (M918T), producing a genotype in *M. domestica* referred to as *super‐kdr* (L1014F + M918T).^4^, ^14^, ^15^ *Super‐kdr* like L1014F + M918I genotypes are found among pyrethroid‐resistant *Cimex hemipterus*.^16^, ^17^, ^18^ Pyrethroid resistance is associated with nonsynonymous mutations in the *vgsc* of aphids.^19^, ^20^, ^21^, ^22^, ^23^ For example, L1014F, M918T and M918L, and their allelic combinations, are found among pyrethroid‐resistant *Myzus persicae* ^24^, ^25^, ^26^ and *Aphis gossypii*.^27^, ^28^ *Super‐kdr* genotypes (L1014F + M918I) were associated with the highest levels of pyrethroid resistance among field‐collected *Aphis glycines*,^29^, ^30^ similar to that reported for *M. persicae*.^31^ The L925F (leucine‐to‐phenylalanine) mutation has been found in several aphid species^32^ and the L925M + L1014F and M918L + L1014F genotypes also are associated with resistant *A. glycines* individuals.^29^

Although mutations that reduce the toxic effects of pyrethroids confer a selective advantage to insects leading to their increased prevalence in field populations, pleiotropy also is observed (i.e. a fitness cost).^33^ Several factors impact the rate of increase and persistence of resistant phenotypes in insect populations, such as the pest management practices used, the initial frequency and selective advantage of resistance alleles, selection pressure, gene flow, and the strength and type of fitness costs.^34^, ^35^, ^36^, ^37^, ^38^, ^39^ Fitness costs are measured as reductions in vigor, survival or reproductive capacity compared to susceptible counterparts in the absence of selection. Among aphids, fitness costs associated with insecticide resistance have been observed as reduced reproductive capacity,^40^, ^41^, ^42^, ^43^ increased vulnerability to natural enemies^41^, ^44^ and reduced overwintering survivorship.^45^ These disadvantages can reduce the rate at which resistant alleles approach fixation within a population. The impact of these disadvantages may be most important at the initial stages of resistance development when associated alleles are at a low frequency and mostly present among heterozygotes.^46^ Allelic dominance also can affect the degree to which fitness costs impact the effects of selection^47^ and the persistence of resistance alleles when selection pressures are relaxed.^48^

Soybean aphid, *A. glycines* (Hemiptera: Aphididae), is an invasive pest of soybean, (*Glycine max*), notably in the United States,^49^ that can reduce soybean yield by ≤40% when left unmanaged.^50^ Foliar applications of pyrethroids are the primary strategy adopted by farmers to manage *A. glycines*,^51^, ^52^ resulting in the increased prevalence of resistant phenotypes in field populations throughout the northcentral USA.^30^, ^53^ If pyrethroid resistance becomes more frequent, reaching 50% of a given field population, the capacity for these insecticides to prevent economic yield loss is predicted to decrease.^52^ Efforts to develop an insecticide resistance management (IRM) plan to prevent this outcome would benefit from understanding the trade‐offs that *A. glycines* may experience in the absence of the selective advantage conferred by pyrethroid resistance.

We conducted a series of experiments to estimate the degree to which fitness costs are related with lambda‐cyhalothrin and bifenthrin resistance associated with different mutations in *vgsc* genes of isofemale lines (i.e. clones). Life table analyses were constructed and used to determine if any differences in fitness occurred among isofemale lines with one or two *vgsc* mutations (M918I, M918L, L925M and L1014F) compared to a susceptible control. The occurrence and magnitude of fitness costs can contribute to our understanding of the future prevalence of different mutations conferring pyrethroid resistance in field populations, and IRM strategies.

## 2 MATERIALS AND METHODS

### 2.1 *Aphis glycines* isofemale lines

*Aphis glycines* were collected from *G. max* plants from fields at Iowa State University (ISU) and University of Minnesota research farms, and one commercial farm (*n* = 5; Table 1). Aphids were collected either before an insecticide application (*n* = 2) or collected two to three days after a foliar application of Warrior II (lambda‐cyhalothrin active ingredient; Syngenta Crop Protection, Greensboro, NC, USA) at a full rate of 0.40 L ha^−1^ (*n* = 3). Live individuals from each location were transported to ISU and maintained on *G. max* plants (Syngenta S24‐K2) without insecticide exposure in separate Percival growth chambers (Percival Scientific, Perry, IA, USA) at 25 ± 2 °C and 50% relative humidity (RH) under a 16 h:8 h, light:dark photoperiod. Plants used to maintain the aphid colonies were grown in a glasshouse (25 ± 2 °C, 50 ± 10% RH, 16 h:8 h, light:dark photoperiod) in 16‐cm diameter plastic pots filled with a soil mixture (SS#1‐F1P, Sungro Horticulture Products, Agawam, MA, USA), watered three times per week and fertilized weekly after emergence (Peters Excel Multi‐Purpose Fertilizer, 21–5‐20 NPK). V3–V4 growth stage plants^54^ were added to the colonies weekly.

**Table 1:** Location, year and insecticide treatment status of isofemale lines

Isofemale line	Location	Year	Application	*vgsc* genotype			
				*M918I*	*M918L*	*L925M*	*L1014F*
SBA‐Boone‐2019‐ISO	Boone, IA	2019	Before	SS	SS	SS	SS
SBA‐Nashua‐2018‐ISO	Nashua, IA	2018	After	SS	SS	SS	RS
SBA‐MN1‐2017‐ISO	Minnesota	2017	After	RS	SS	SS	RS
SBA‐Kanawha‐2019‐ISO	Kanawha, IA	2019	After	SS	SS	SS	RR
SBA‐Darwin‐2019‐ISO	Darwin, MN	2019	Before	SS	RS	RS	SS

Nonsynonymous (amino acid changing) mutations predicted for *vgsc* genotype as determined by Sanger sequencing. # SBA‐ MN1‐2017 sequence data from Valmorbida *et al*.^30^

After ≥25 generations (all through asexual reproduction), a single clonal female was selected randomly from each colony and used to initiate an isofemale line, propagated in growth chambers as described above. Initial females of each isofemale line were propagated by parthenogenesis, whereby each consisted of clonal daughters that were used for further analyses. Naming of each *A. glycines* (soybean aphid, SBA) line indicated the location and year, and initiation from a single individual female (isofemale line, ISO); for example, SBA‐Darwin‐2019‐ISO. For brevity, ISO was removed throughout the text as every line in this study was derived from single individuals.

### 2.2 Sequencing of *vgsc* genes

Mutations in the *vgsc* genes previously associated with pyrethroid resistance in *A. glycines* ^29^, ^30^ were detected in each isofemale line by direct Sanger sequencing. Specifically, fragments from the *A. glycines vgsc* DIIS4–6, DIIIS6–DIVS1 and DIVS4–S6 were amplified by polymerase chain reaction (PCR) and products sequenced as described previously.^30^ In brief, PCR amplification of these three fragments was performed each isofemale line (Table 1), except SBA‐MN1‐2017 which had been characterized previously.^30^ Genomic DNA was isolated separately from individual clones using QuickExtract™ DNA Extraction Solution (Lucigen, Middleton, WI, USA) according to the manufacturer’s instructions, except that the per‐sample volume was adjusted to 50.0 μL. Each fragment was amplified from four independently extracted replicates of each isofemale line. PCR products were purified and bidirectional Sanger sequence data generated on an ABI3700 (Applied Biosystems, Forest City, CA, USA) at the Iowa State University DNA Facility (Ames, IA). Inter‐ and intraspecific variation in individual Sanger reads was predicted using the application tracy ^55^ by alignment to the genomic reference (gene model AG6007485.1 from the *A. glycines* genome assembly Ab_bt1_v6.0),^56^ where co‐occurrence of electropherogram peaks at a nucleotide position were detected using default parameters and defined as putative heterozygotes.

### 2.3 Pyrethroid toxicity bioassays

Bioassays to assess the susceptibility of each isofemale line to pyrethroids were performed between June and September 2020. We used two common commercially used active ingredients, bifenthrin (Type I) and lambda‐cyhalothrin (Type II), that also were used previously in laboratory bioassays to estimate levels of resistance among field‐collected *A. glycines*.^30^, ^53^, ^57^ Type I pyrethroids do not have a cyano moiety at the α‐position and are characterized by symptoms such as hyperactivity, uncoordination response to a single stimulus, and finally paralysis. Type II compounds have an α‐cyano moiety, causing a pronounced convulsive phase, with membrane depolarization and suppression of the action potential.^11^, ^58^ We performed these bioassays with lambda‐cyhalothrin (97.7% purity; Control Solutions Inc., Pasadena, CA, USA) and bifenthrin (98% purity; Chem Service Inc., West Chester, PA, USA) using a leaf‐dip bioassay following recommendations by the Insecticide Resistance Action Committee (IRAC) for detecting resistance.^59^ For this, stock solutions of lambda‐cyhalothrin and bifenthrin were prepared separately using acetone, and then diluted with 0.05% (v/v) Triton X‐100 (Alfa Aesar, Tewksbury, MA, USA) in distilled water to prepare treatment concentrations (0.056–56 μg mL^−1^). The control treatment consisted of 0.05% (v/v) Triton X‐100 in distilled water, and acetone (<0.05%) equal to the concentration in the treatment with the highest concentration of lambda‐cyhalothrin or bifenthrin.

Leaves from the first and second trifoliate of *G. max* at V3–V4 growth stage^54^ were cut in 3.8‐cm diameter disks using a hole punch (Fiskars, Helsinki, Finland). The disks were submerged individually in one of the treatment solutions with gentle agitation for 10 s and air‐dried with abaxial side up on a paper towel. Plastic cups (29.6‐mL, Choice Paper Company, New York, NY, USA) were filled with ~20 mL of 1% w/v agar (Bacto™ Agar; Becton, Dickinson and Co., Franklin Lakes, NJ, USA). Leaf disks were placed abaxial side down onto the agar surface before congealing. A droplet of distilled water was added to the agar bed to increase leaf disk adherence when needed. Each leaf disk was infested with 20 apterous mixed‐age adult aphids collected from *G. max* plants using paintbrushes, with each treatment concentration performed across three replicate leaf disks (triplicate; *n* = 60 aphids total). Plastic cups were sealed with a close‐fitting ventilated lid, and incubated in a growth chamber (25 ± 2 *°*C, 70% RH, 16 h:8 h, light:dark photoperiod). Mortality was assessed 48 h post‐treatment, and aphids were considered moribund if unable to right themselves after 10 s.^57^, ^59^

Mortality data were used to estimate slope and the LC_50_ of each isofemale line using a three‐parameter log‐logistic function of the drc package,^60^ implemented in R v3.6.2, and LC_50_ values were considered significantly different if 95% confidence intervals (CIs) did not overlap.^61^ The resistance ratio (RR) was calculated relative to the susceptible line (SBA‐Boone‐2019) for each of the other populations following methods described previously.^62^ The LC_50_ values of SBA‐Nashua‐2018, SBA‐MN1‐2017, SBA‐Kanawha‐2019 and SBA‐Darwin‐2019 were divided by the LC_50_ of the susceptible isofemale line (SBA‐Boone‐2019).

### 2.4 Life table analysis

Life history parameters from each isofemale line were measured in July and August 2020. From each *A. glycines* isofemale line, 45 apterous mixed‐age adult aphids were transferred individually onto an untreated V2–V3 stage *G. max* leaflet placed into a Petri dish containing a moistened filter paper at the bottom. Petri dishes then were sealed with parafilm to prevent aphid escape and stored in a growth room at 25 ± 2 *°*C and 70% RH, under a 16 h:8 h, light:dark photoperiod. After 24 h, newly emerged nymphs (<24‐h‐old) were transferred to new leaflets and maintained individually in a Petri dish as described above. A total of 45 nymphs were transferred for SBA‐Boone‐2019, SBA‐Nashua‐2018, SBA‐MN1‐2017 and SBA‐Darwin‐2019 isofemale lines, and 43 for SBA‐Kanawha‐2019. Leaflets were replaced every seven days and the filter paper was moistened as needed.^63^ *Aphis glycines* morphological characteristics were used to determine developmental stages^64^, ^65^ along with the presence of exuviae, which were removed once detected. The developmental stages, fecundity and adult longevity were measured daily until the death of the aphid. The offspring were counted and removed daily.

Life table analysis was performed according to the age‐stage, two‐sex life table theory,^66^, ^67^ within the TWO‐SEX mschart program.^68^ Biological and demographic parameters were calculated according to Chi and Liu^66^ and Chi.^67^ The Bootstrap procedure^69^with 100 000 replicates^70^ was used to estimate means and standard errors (SEs) of each parameter. We used a paired bootstrap test at the 95% significance level within TWO‐SEX mschart ^68^ to determine differences for each biological and demographic parameter among the five isofemale lines. We used Kaplan–Meier to estimate survival curves and log‐rank tests to compare survival curves between isofemale lines. The R packages survival ^71^and surviminer ^72^ were used for the survival analysis.

## 3 RESULTS

### 3.1 Detection of mutations through sequencing of *vgsc* genes

**Figure 1:**
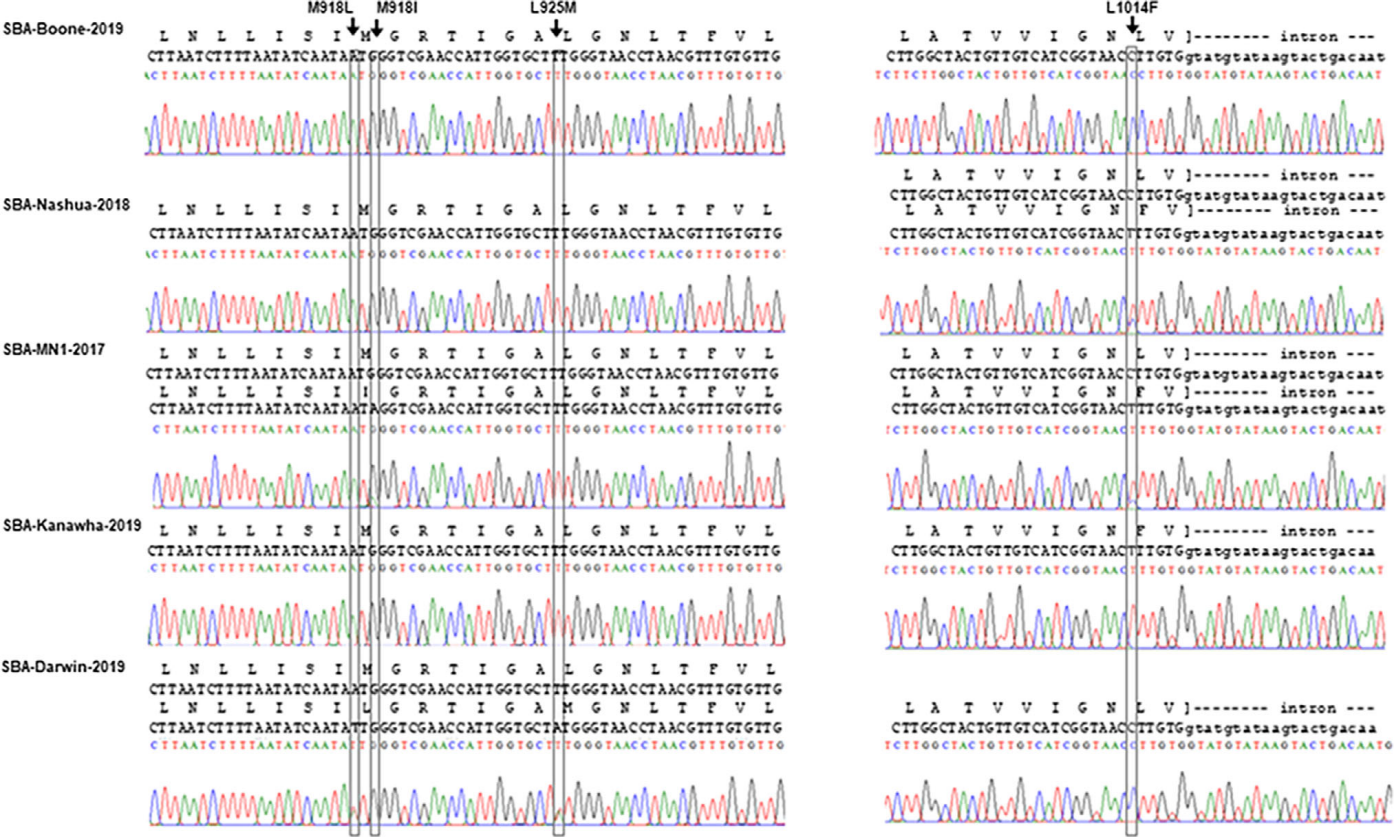
Electropherogram from Sanger sequence reads from the *A. glycines vgsc* gene. Substitution mutations predicted to cause amino acid changes M918I, M918L, L925M and L1014F in translated amino acid sequence in one or more pyrethroid‐resistant *A. glycines* isofemale lines are indicated by arrows. Heterozygote genotype present at co‐occurring nucleotide signals in SBA‐Nashua‐2018 for the L1014F mutation, SBA‐MN1‐2017 for M918I and L1014F mutations, and in SBA‐Darwin‐2019 for M918L and L925M mutations. Data from SBA‐Kanawha‐2019 indicate homozygosity for the mutant 1014F allele. By contrast, these mutations are not predicted in the susceptible SBA‐Boone‐2019 isofemale line. Multiple sequence alignment of the entire sequenced fragment shown in Fig. S1.

Direct Sanger sequencing of DIIS4–6, DIIIS6–DIVS1 and DIVS4–S6 regions of the *A. glycines vgsc* genes (GenBank accessions OL321811–321825) revealed a total of eight mutations within and between the four pyrethroid‐resistant isofemale lines compared to the reference genome. Four of the eight mutations were nonsynonymous (amino acid changing). No mutations were predicted in sequence data for the pyrethroid susceptible line SBA‐Boone‐2019. There were no differences among the four replicate reads from each line. Specifically, comparisons within and between resistant isofemale lines predicted four nonsynonymous (amino acid changing) mutations in the DIIS4–S6 fragment electropherogram results: an A‐to‐T transversion at AG6007485‐RA position 2782 causing the M918L mutation; a G‐to‐A transition at AG6007485‐RA position 2784 causing the M918I mutation; aT‐to‐A transversion at AG6007485‐RA position 2803 causing the L925M mutation; and a C‐to‐T transition at AG6007485‐RA position 3070 resulting in a predicted L1014F knockdown resistance (*kdr*) mutation (Fig. 1). Analyses also predicted either single incidence (homozygosity) or co‐occurrence of respective nucleotide signals (heterozygosity) for M918I, M918L, L925M and L1014F in electropherogram data from the pyrethroid‐resistant clones (Fig. 1).

Considering these sequence results, genotypes for SBA‐Nashua‐2018 and SBA‐Kanawha‐2019 were heterozygous and homozygous, respectively, for the L1014F mutation. SBA‐Darwin‐2019 was heterozygous for M918L and L925M mutations, and SBA‐MN1‐2017 was heterozygous for both the M918I and L1014F mutations (e.g. *super‐kdr* genotype). The SBA‐Boone‐2019 isofemale line was wild‐type (WT) at all loci (Table 1; Fig. S1).

Sequence data from the DIIIS6‐DIVS1 region had three of the eight total predicted mutations. Two involved transitions between purine (R) nucleotides (A and G) and one a transversion between T and G nucleotides (K), of which all were in a 3rd position (synonymous or nonamino acid changing; Fig. S2, remaining invariant positions of alignment not shown). Furthermore, all lines were putatively heterozygous for these mutations, with the exception for the glycine 1545 codon in the SBA‐Kanawha‐2019 that was homozygous for the WT allele. These synonymous changes were located in or immediately downstream of the region encoding the DIII S6 helix (Fig. S3). Comparisons within the Sanger sequenced DIVS4‐S6 fragment predicted a single substitution mutation. This was either A nucleotide or co‐occurring A and G nucleotide signals in the 3rd position of codon encoding a leucine residue (Fig. S4, remaining invariant positions of alignment not shown). Specifically, SBA‐Nashua‐2018, SBA‐MN1‐2017 and SBA‐Kanawha‐2019 were heterozygous with co‐occurring purine (R) A and G nucleotides. This silent mutation was located upstream of DIVS6 (Fig. S5).

### 3.2 Susceptibility to pyrethroids

Leaf‐dip bioassays showed a range of susceptibilities to lambda‐cyhalothrin and bifenthrin among the isofemale lines containing one or more *vgsc* mutations associated with pyrethroid resistance. Specifically, SBA‐Darwin‐2019 showed the highest LC_50_ estimated for both lambda‐cyhalothrin [10.90 (8.71–13.09) μg mL^−1^] and bifenthrin [12.40 (10.19–14.61) μg mL^−1^]. SBA‐Nashua‐2017 had the lowest estimated LC_50_ for both lambda‐cyhalothrin [0.90 (0.64–1.16) μg mL^−1^], and bifenthrin [1.29 (0.93–1.66) μg mL^−1^]. The LC_50_ concentrations estimated for SBA‐Nashua‐2017, SBA‐MN1‐2017, SBA‐Kanawha‐2019 and SBA‐Darwin‐2019 were significantly higher compared to SBA‐Boone‐2019 for both insecticides (Table 2). These results further showed that the estimated LC_50_ was significantly lower for SBA‐Nashua‐2017 compared with all other isofemale lines (SBA‐MN1‐2017, SBA‐Kanawha‐2019 and SBA‐Darwin‐2019) for both insecticides. The estimated LC_50_ was significantly higher for SBA‐Darwin‐2019 compared with all other isofemale lines for bifenthrin. The SBA‐Boone‐2019 isofemale line was considered susceptible, due to its estimated LC_50_ to lambda‐cyhalothrin [0.29 (0.23–0.35) μg mL^−1^] and bifenthrin [0.23 (0.18–0.28) μg mL^−1^] (Table 2). The calculated RR varied ≥9.6‐fold across the five *A. glycines* isofemale lines when compared to our standard susceptible line, SBA‐Boone‐2019, when exposed in leaf‐dip bioassays to lambda‐cyhalothrin (RR range 3.10 to 37.58) and bifenthrin (5.60 to 53.91; Table 2).

**Table 2:** Toxicity of lambda‐cyhalothrin and bifenthrin to *A. glycines* isofemale lines

	Lambda‐cyhalothrin						
Isofemale line	*n*	Slope ± SE	LC_50_ (95% CI)*	RR^†^	*χ* ^2^ (d.f.)	*P*‐value	Group^‡^
SBA‐Boone‐2019‐ISO	480	1.36 ± 0.16	0.29 (0.23–0.35) a	‐	6.74 (5)	0.240	R0^L^
SBA‐Nashua‐2018‐ISO	480	1.48 ± 0.27	0.90 (0.64–1.16) b	3.10	4.12 (5)	0.531	R1^L^
SBA‐MN1‐2017‐ISO	480	1.59 ± 0.21	10.33 (7.70–12.96) c	35.62	1.43 (5)	0.920	R2^L^
SBA‐Kanawha‐2019‐ISO	480	2.26 ± 0.32	10.75 (8.51–13.00) c	37.06	6.55 (5)	0.256	R2^L^
SBA‐Darwin‐2019‐ISO	480	2.35 ± 0.34	10.90 (8.71–13.09) c	37.58	3.25 (5)	0.661	R2^L^
**Bifenthrin**							
SBA‐Boone‐2019‐ISO	420	2.77 ± 0.54	0.23 (0.18–0.28) a	‐	5.29 (4)	0.258	R0^B^
SBA‐Nashua‐2018‐ISO	420	1.90 ± 0.46	1.29 (0.93–1.66) b	5.60	5.61 (4)	0.229	R1^B^
SBA‐MN1‐2017‐ISO	420	3.41 ± 0.49	7.38 (6.31–8.45) c	32.08	1.22 (4)	0.873	R2^B^
SBA‐Kanawha‐2019‐ISO	420	2.20 ± 0.29	7.26 (5.84–8.69) c	31.56	8.66 (4)	0.070	R2^B^
SBA‐Darwin‐2019‐ISO	480	2.98 ± 0.51	12.40 (10.19–14.61) d	53.91	3.80 (5)	0.577	R3^B^

* LC_50_ values followed by different letters within a column are significantly different from each other through nonoverlap of 95% CI.

^†^ Resistance Ratio (RR), LC_50_ of a clonal lineage divided by the LC_50_ of the susceptible lineage (SBA‐Boone‐2019‐ISO).

^‡^ *Aphis glycines* isofemale lines with LC_50_ estimates that are not significantly different from one another.

Based on these nonoverlapping LC_50_ estimates and associated RR for each isofemale line, we defined those considered susceptible and to have significantly different levels of resistance from lambda‐cyhalothrin and bifenthrin bioassays (Table 2). Specifically for lambda‐cyhalothrin, we defined a susceptible group (R0^L^) comprising only SBA‐Boone‐2019, and used this group to define resistance in the three other groups. The first significant incremental increase in resistance is observed in the group consisting of only SBA‐Nashua‐2019 (R1^L^). The second group (R2^L^) consisted of SBA‐MN1‐2017, SBA‐Kanawha‐2019 and SBA‐Darwin‐2019 which have a significantly higher LC_50_ compared to R0^L^ and R1^L^, but no differences among each other. We used a similar nomenclature for these populations when exposed to bifenthrin in the leaf‐dip bioassays. Correspondingly, four phenotypes with significantly different LC_50_ were defined (Table 2), with SBA‐Darwin‐2019 in the R3^B^ group having the highest level of resistance.

### 3.3 Life table parameters of susceptible and pyrethroid‐resistant aphids

Time spent in the N2 and N4 developmental stages, adult pre‐ovipositional period, total pre‐ovipositional period, as well as adult longevity and number of offsprings varied significantly among the five isofemale lines. Significant differences in nymphal development time and reproduction were not consistent among pyrethroid‐resistant isofemale lines when compared to the susceptible line (SBA‐Boone‐2019; Table 3). For example, the mean days in N2 were significantly lower for all lines compared to the susceptible aphids, but the corresponding mean number of days in N4 was significantly lower only between SBA‐Darwin‐2019 and all other lines. No differences were observed when comparing any of the N1 or N3 stages. The shortest adult pre‐oviposition and total pre‐oviposition periods were observed in SBA‐MN1‐2017, which also had the longest oviposition period and greatest adult longevity of all others except SBA‐Darwin‐2019 (Table 3). Fecundity also was significantly greater for SBA‐MN1‐2017 and SBA‐Darwin‐2019 compared to the other isofemale lines.

**Table 3:** Biological parameters of pyrethroid‐susceptible and ‐resistant *A. glycines* isofemale lines

Biological parameter	Isofemale line				
	SBA‐Boone‐2019‐ISO	SBA‐Nashua‐2018‐ISO	SBA‐MN1‐2017‐ISO	SBA‐Kanawha‐2019‐ISO	SBA‐Darwin‐2019‐ISO
N1 (days)	1.26 ± 0.06a	1.26 ± 0.06a	1.24 ± 0.06a	1.20 ± 0.06a	1.33 ± 0.07a
N2 (days)	1.33 ± 0.07a	1.20 ± 0.06ab	1.13 ± 0.05b	1.29 ± 0.07ab	1.26 ± 0.06ab
N3 (days)	1.22 ± 0.06a	1.18 ± 0.05a	1.25 ± 0.06a	1.17 ± 0.06a	1.32 ± 0.07a
N4 (days)	1.29 ± 0.02a	1.40 ± 0.07a	1.44 ± 0.07a	1.46 ± 0.08a	1.12 ± 0.05b
APOP	0.23 ± 0.06a	0.17 ± 0.05a	0.07 ± 0.000b	0.24 ± 0.08a	0.22 ± 0.07a
TPOP	5.32 ± 0.071a	5.14 ± 0.081a	5.09 ± 0.000b	5.29 ± 0.100a	5.25 ± 0.077a
Oviposition period (days)	10.62 ± 0.55b	8.58 ± 0.77c	12.64 ± 0.52a	11.32 ± 0.71ab	12.40 ± 054a
Adult longevity (days)	17.71 ± 0.84ab	15.44 ± 1.03b	19.71 ± 0.82a	17.60 ± 1.16ab	19.40 ± 0.98a
Fecundity	43.11 ± 2.49bc	35.16 ± 3.59c	52.00 ± 2.46a	43.53 ± 3.42bc	48.09 ± 2.62ab

Mean ± SE was estimated using 100 000 bootstrap replications. Different letters within the same row indicate significant differences among the clonal lines at the *P* < 0.05 level, with a paired bootstrap test. APOP, adult pre‐oviposition period; TPOP, total pre‐oviposition period.

The demographic parameters reveal that the SBA‐MN1‐2017 had greater overall fitness (Table 4). Specifically, finite rate of increase and intrinsic rate of increase were significantly higher for SBA‐MN1‐2017 compared to all others, including the three lines with increased levels of resistance. Furthermore, SBA‐MN1‐2017 had significantly higher net reproductive rate than SBA‐Nashua‐2018 and SBA‐Kanawha‐2019, and significantly higher gross reproductive rate than SBA‐Darwin‐2019.

**Table 4:** Demographic parameters of pyrethroid‐susceptible and ‐resistant *A. glycines* isofemale lines

Demographic parameter	Isofemale line				
	SBA‐Boone‐2019‐ISO	SBA‐Nashua‐2018‐ISO	SBA‐MN1‐2017‐ISO	SBA‐Kanawha‐2019‐ISO	SBA‐Darwin‐2019‐ISO
Net reproductive rate (*R*_o_)	41.15 ± 2.61ab	32.80 ± 3.59c	49.68 ± 2.84a	39.48 ± 3.65bc	43.82 ± 3.13ab
Finite rate of increase (*λ*, d^−1^)	1.46 ± 0.00b	1.45 ± 0.01b	1.49 ± 0.01a	1.44 ± 0.01b	1.45 ± 0.01b
Intrinsic rate of increase (*r*, d^−1^)	0.38 ± 0.00b	0.37 ± 0.01b	0.40 ± 0.00a	0.36 ± 0.00b	0.37 ± 0.00b
Mean generation time (*T*, days)	9.77 ± 0.11ab	9.30 ± 0.17c	9.65 ± 0.10bc	9.97 ± 0.14ab	9.99 ± 0.10a
GRR	52.97 ± 2.06ab	52.40 ± 3.94ab	57.52 ± 1.44a	54.23 ± 1.60ab	53.19 ± 1.42b

Mean ± ‐SE)‐ was estimated using 100 000 bootstrap replications. Different letters within the same row indicate significant differences among the isofemale lines at *P* < 0.05 level, with a paired bootstrap test. GRR, gross reproductive rate.

**Figure 2:**
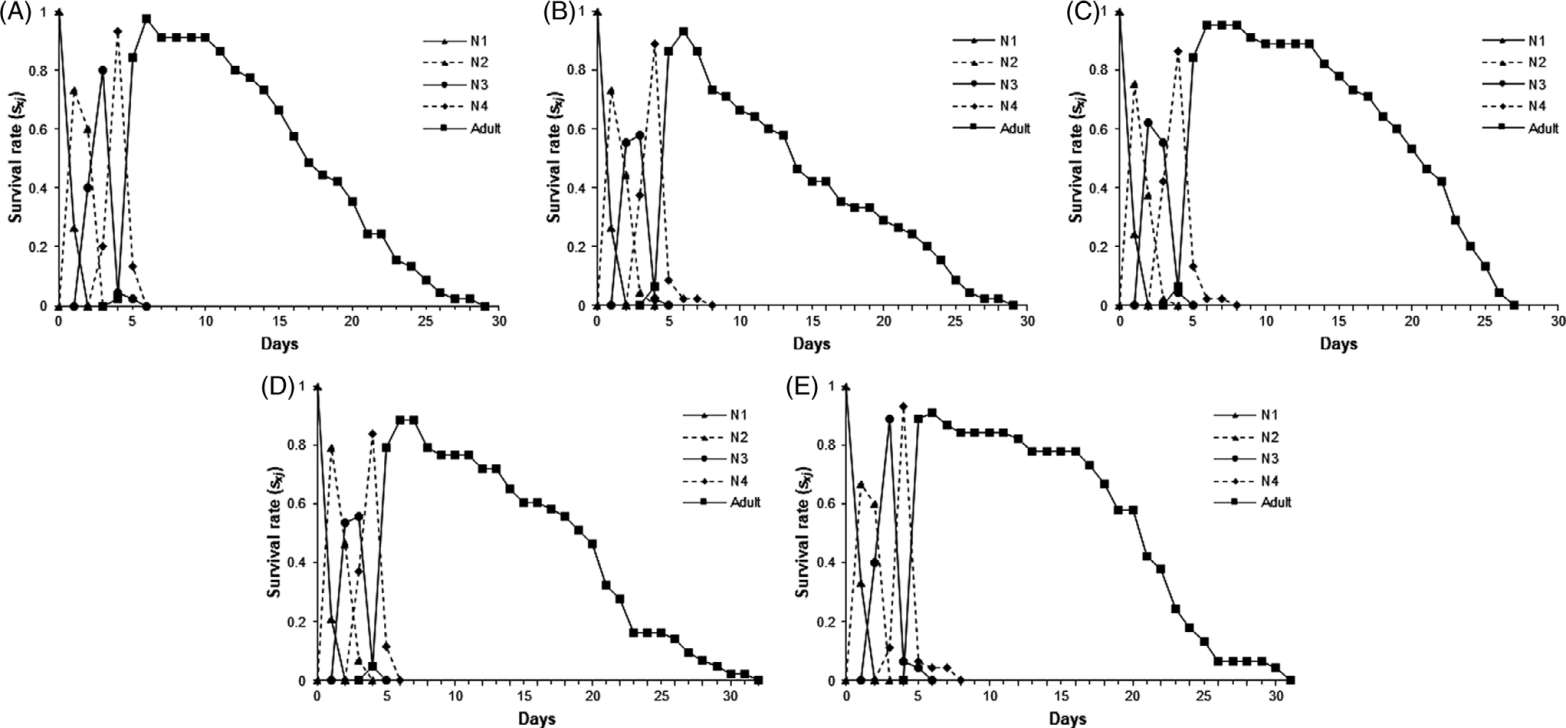
Age‐stage specific survival rate (*s*_*xj*_) in female isolines: (A) SBA‐Boone‐2019; (B) SBA‐Nashua‐2018; (C) SBA‐MN1‐2017; (D) SBA‐Kanawha‐2019; and (E) SBA‐Darwin‐2019.

**Figure 3:**
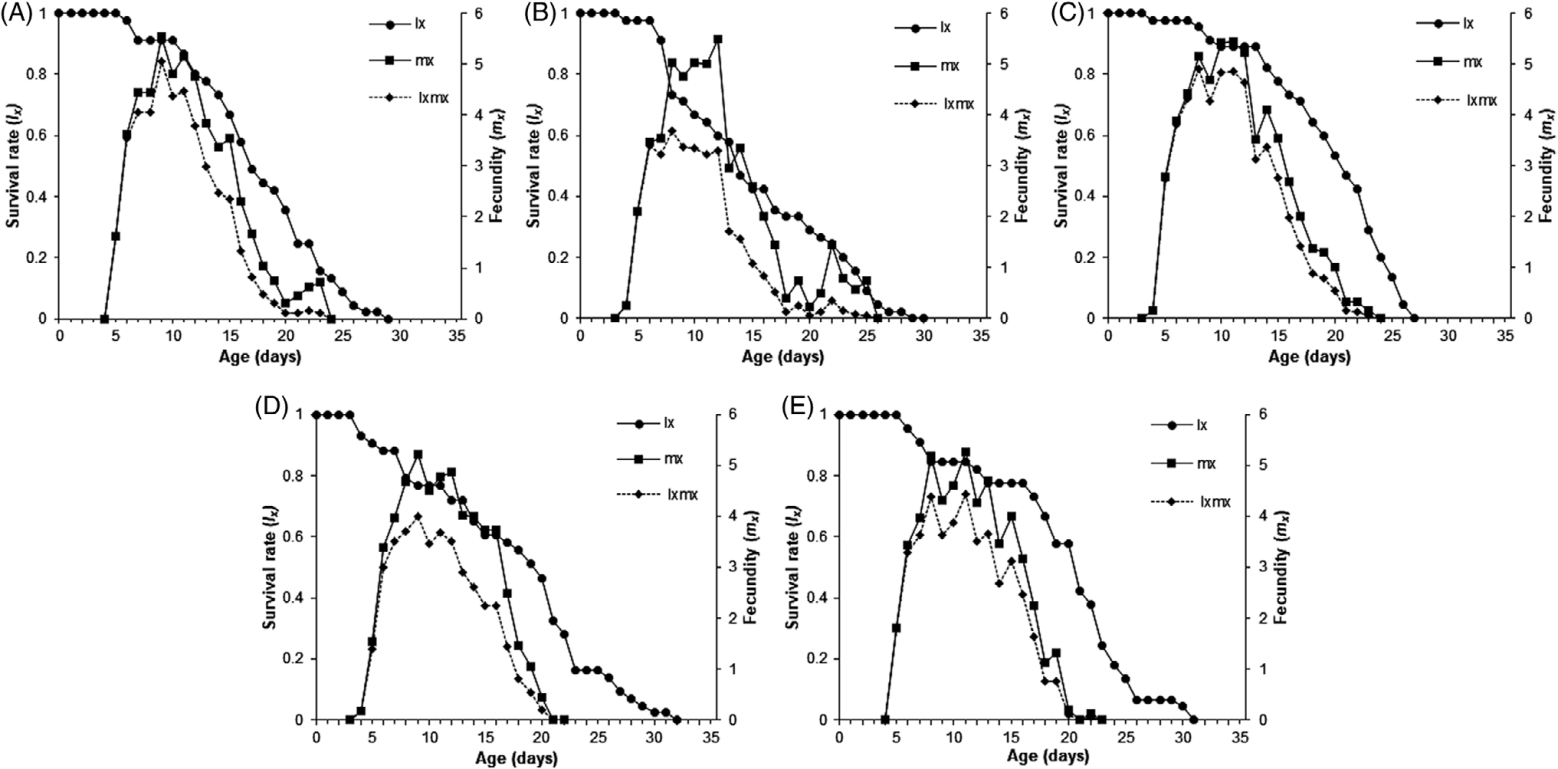
Age‐specific survival rate (*l*_*x*_), age‐specific fecundity (*m*_*x*_) and age‐specific maternity (*l*_*x*_ *m*_*x*_) of pyrethroid‐susceptible and ‐resistant *A. glycines* isolines: (A) SBA‐Boone‐2019; (B) SBA‐Nashua‐2018; (C) SBA‐MN1‐2017; (D) SBA‐Kanawha‐2019; and (E) SBA‐Darwin‐2019.

The lowest net reproductive rate was estimated for SBA‐Nashua‐2018, whereas none of the remaining demographic parameters, with the exception of mean generation time, were significantly different from the susceptible line (Table 4). The age‐specific survival rates (*s*_*xj*_) overlapped among the developmental stages (N1–N4; Fig. 2). The earliest and greatest decline in adult female survival occurred after Day 8 for SBA‐Nashua‐2018, whereas analogous declines occurred for the other isofemale lines at Day 12. Likewise, SBA‐Nashua‐2018 had a lower survival (*l*_*x*_) and net maternity (*l*_*x*_ *m*_*x*_) rates. Net maternity peaks were highest for SBA‐Boone‐2019, SBA‐MN1‐2017 and SBA‐Darwin‐2018 isofemale lines (Fig. 3). Kaplan–Meier survival curves and log‐rank tests showed differences in the survival probability among the isofemale lines (Fig. S6). Significant pairwise differences were observed between SBA‐Nashua‐2018 and SBA‐Darwin‐2019 (*P* = 0.036). No significant differences in survival probability were observed for any other comparisons.

## 4 DISCUSSION

Differences in fitness among individuals in a population can impact their relative abundance and genetic contribution to future generations. When under selection, traits such as insecticide resistance are advantageous and genotypes conferring these phenotypes can rapidly increase in a population.^73^ The persistence and overall success of these adaptations are dependent upon an interplay between selective advantage and any detrimental effects of the associated mutations on fitness (e.g. fitness costs).^74^ Resistance generally is considered to have associated fitness costs when individuals are competing in the absence of the insecticide.^33^, ^46^ However, the presence of modifier genes^75^, ^76^ and mechanisms to stop the production of detoxification enzymes in the absence of the selection agent^77^ can ameliorate fitness in resistant individuals. One or more nonsynonymous mutations in the *vgsc* are associated with *A. glycines* genotypes possessing varying levels of pyrethroid resistance.^29^, ^30^ This study demonstrates that these genetically distinct lines carry different levels of relative fitness in absence of pyrethroid exposure.

Among the five *A. glycines* isofemale lines initiated from collections made in Iowa and Minnesota soybean fields, we identified unique genotypes for each based on a combination of mutations in the DII S4–S6 region of the *vgsc* gene (Table 1). Amongst these genotypes, we defined phenotypic groups categorized by levels of resistance to lambda‐cyhalothrin and bifenthrin (Table 2). The range of resistance that we observed is analogous to estimates from studies published previously,^30^, ^57^ revealing the presence of phenotypic variations within and between field locations. Although each of these mutations conferring the varied levels of resistance have arisen independently (e.g. at different loci of the *vgsc* gene), it remains unknown if resistance alleles arose *de novo* since or were extant within the population before widespread pyrethroid use.^73^, ^78^ Any direct implication of different amino acid changes encoded by the genotypes in the four isofemale lines in our study as completely causal of corresponding levels of lambda‐cyhalothrin and bifenthrin resistance remains speculative. This is especially true given the potential for a portion of these resistance traits to be conferred by detoxification enzymes^79^ or by interactions among *vgsc* mutations that alter pyrethroid binding in a nonadditive fashion.^80^ Although previous work showed that SBA‐MN1‐2017 (heterozygous *super‐kdr* M918I + L1014F) did not present cross‐resistance and the exposure to detoxification enzyme inhibitors did not affect its susceptibility to lambda‐cyhalothrin,^30^ the genetic or biochemical basis for estimated differences in the level of resistance and cross‐resistance for the other isofemale lines remains unknown pending further investigations.

Our results suggest that different nonsynonymous mutations in the *vgsc* might confer similar levels of resistance. For example, the heterozygous *super‐kdr* (L1014F + M918I) of SBA‐MN1‐2017, homozygous *kdr* of SBA‐Kanwha‐2019 and heterozygous L925M + M918L of SBA‐Darwin‐2019, had similar LC_50_ when exposed to lambda‐cyhalothrin (Group R2^L^; Table 2). The SBA‐Nashua‐2018 (heterozygous, RS, for the L1014F *kdr* mutation) had the lowest LC_50_ for lambda‐cyhalothrin and bifenthrin compared with homozygous L1014F (SBA‐Kanawha‐2019) and heterozygous *super‐kdr* M918I + L1014F (SBA‐MN1‐2017; Table 2). These two comparisons agree with prior evidence that homozygous L1014F genotypes enhance aphid resistance to pyrethroids compared to heterozygous genotypes,^13^, ^81^ and the increased resistance of *super*‐*kdr* genotypes in aphids^82^ and other insects.^4^ Additionally, the presence of different mutations giving rise to similar and potentially field‐relevant levels of resistance is challenging in the context of IRM monitoring programs using genetic markers, where phenotypic effects of allele combinations probably need to be considered.

The L925M + M918L genotype (SBA‐Darwin‐2019) showed the highest level of bifenthrin resistance (group R3^B^; Table 2), but no significant increase in resistance to lambda‐cyhalothrin compared with SBA‐Kanawha‐2019, and SBA‐MN1‐2017 (group R2^L^; Table 2). Individual or combinations of *vgsc* DIIS4–S6 target site mutations in resistant insect genotypes may also differentially affect the interaction with type I (e.g. bifenthrin) and type II pyrethroids (e.g. lambda‐cyhalothrin).^43^, ^82^, ^83^, ^84^ Our data suggest that an increase in bifenthrin resistance in SBA‐Darwin‐2019 could be a consequence of unique changes in the interactions between type I pyrethroids and a binding pocket with amino acids leucine and methionine at the 918 and 925 positions, respectively. Pyrethroids may have a dual binding site, including the lipid interface in the DIIS4 to S5 linker region and a second putative receptor site in both S6DI and S6DII.^84^, ^85^ Specific differences in any change of lambda‐cyhalothrin or bifenthrin to the *vgsc* L925M + M918L variant of SBA‐Darwin‐2019 remains speculative and requires further testing. Replicated trials of independent isofemale lines with the same *vgsc* genotype could improve our understanding how each mutation accounts for the levels of pyrethroid resistance observed in *A. glycines* in North America, or if other genetic factors are involved.

Given these caveats regarding the impact of the *vgsc* mutations on the observed phenotypes, we elected to focus on these five isolines as they allowed for an initial exploration of the impact of pyrethroid resistance on *A. glycines* fitness. We initially hypothesized that variation across the genotypes and phenotypes of *A. glycines* would produce a range of life‐history parameters revealing that resistance to pyrethroids is associated with a decline in fitness. We predicted that isolines with mutations in the *vgsc* gene would negatively affect fitness. Our results did not reveal a trend across any of the various parameters measured to suggest that the susceptible isoline consistently outperformed the various resistant isolines (Tables 3 and 4). This may not be surprising given that fitness varies across different unrelated genetic backgrounds,^86^, ^87^, ^88^ or lines with other mechanisms or *vgsc* mutations conferring pyrethroid resistance.^89^ Likewise, no clear association of reduced reproductive performance and insecticide resistance were shown among clones of *M. persicae*.^90^, ^91^, ^92^ Regardless, our data indicate a significant reproductive advantage of one isofemale line carrying heterozygous *super*‐*kdr* M918I + L1014F genotype (Table 3), observed in pre‐oviposition period and increased overall fecundity. Likewise, a significant higher reproductive performance was observed in insecticide‐resistant *M. persicae* ^90^ and *Sitobion avenae*.^93^

Although our results suggest that mutations in the *vgsc* genes of *A. glycines* did not confer a fitness cost, limitations within this study prevented us from reaching this conclusion. First, we lack a full complement of possible genotypes for the various *vgsc* mutations (i.e. SS, RS and RR). It is possible that the missing genotypes (e.g. RR for M918I, M918L and L925M) and their combinations suffer a reduction in some parameters measured within the life table analysis. Such a reduction may help explain their absence from our samples. Second, variation in the genetic background of the five clonal isofemale lines used in this study may have prevented us from observing fitness costs associated with resistance. Specifically, selection for other biotic or abiotic factors unrelated to pyrethroid resistance might be responsible for the observed increased fitness of SBA‐MN1‐2017. Third, our observations were conducted under laboratory conditions and we did not perform density‐dependent experiments to explore changes in the frequency of resistant alleles over time. Freeman *et al* .^33^ suggest that the association of fitness costs with resistance should be measured within congenic lines and include multiple measurements across different assays. Although highly useful, various challenges exist for generating aphid congenic lines through backcrossing as a result of their clonal nature and the low efficiency in generating outcrossed individuals.^82^, ^94^ Future studies involving replicated independent lines from different locations each carrying the same *vgsc* genotype in a diverse genetic backgrounds may address the potential influence of other genetic loci on fitness parameters measured in the *super*‐*kdr* genotype of SBA‐MN1‐2017.

There are several points within the life history of *A. glycines* when a fitness cost could be experienced beyond what we modelled in our life table analysis. The frequency of resistant alleles might decline during the summer in the absence of the selection agent, when aphids migrate to their overwinter host, *Rhamnus cathartica* (buckthorn) to reproduce sexually, and during the migration to soybean fields in the following spring. Measurements of several parameters under different environmental conditions that reflect the complex life history of *A. glycines*, including frequency and survival of resistant alleles in the overwintering host, may be necessary for understanding the persistence of pyrethroid resistance in *A. glycines*. For now, our results suggest that in the simplest scenario modelled by the life table analysis, fitness costs were not observed.

Despite the limitations of our study, it does provide some insight into the potential of fitness cost associated with pyrethroid‐resistant *A. glycines*, and their possible impacts on the evolution of resistance to pyrethroids in field populations. In general, a fitness cost is expected to delay the fixation of resistance within a population by reducing an increase in the subpopulation with the corresponding genotype.^36^, ^88^, ^95^, ^96^, ^97^ Understanding the occurrence and impacts of fitness costs are essential in developing and implementing IRM programs.^88^, ^98^ If pleotropic effects were associated with pyrethroid‐resistant soybean aphids, using an insecticide with a different mode of action would be expected to decrease the frequency of resistant individuals. If this strategy is adopted, farmers could not only manage a resistant population, but also prevent the single mode selection and spread of pyrethroid‐resistant aphids. Such strategies would require farmers to increase the adoption of IPM and IRM programs throughout the northcentral USA. Regardless, switching to an alternative insecticide is challenging in the United States. Other active ingredients are more costly,^52^ and one of the more commonly used active ingredients (chlorpyrifos) recently was banned by the Environmental Protection Agency.^99^ Switching to another form of pest management (e.g. aphid‐resistant varieties) is possible and cost‐effective, but is limited to varieties that are not currently glyphosate‐resistant which can require a substantial change to a farmer’s weed management plan.^52^

In absence of data revealing a pleotropic effect, models and predictions of resistance spreading should not assume a fitness cost. As demonstrated in our study, the opposite is possible. Note that resistance confers an increase in fitness, but this trait could be within a genetic back‐ground that has greater fitness than a susceptible/WT subpopulation which, in turn, could explain a relatively sudden increase in a resistant population. However, this is likely stochastic and challenging to model.

## 5 CONCLUSIONS

Field‐evolved resistance to pyrethroids highlights the need to adopt strategies to mitigate the effects of pyrethroid resistance and delay resistance evolution to other chemistries. To date, laboratory‐selected pyrethroid‐resistant *A. glycines* have presented cross‐resistance,^100^ yet this was not present in a field‐collected resistance population,^30^ suggesting insecticides with different modes of action can still be used to manage outbreaks of *A. glycines*. Although our experiments were performed under laboratory conditions, the observed high levels of resistance associated with increased reproductive performance are concerning and require management strategies to prevent these clones from thriving throughout the growing season. Further studies on the distribution and consequences of field‐evolved resistance to pyrethroids are needed to reduce the selection pressure and maintain the use of these insecticides for *A. glycines* IPM programs throughout the northcentral United States.

## Supporting information


**Figure S1** Multiple sequence alignment of *A. glycines vgsc* gene fragments encoding predicted α‐helical structures of domain II segment 5 (DII S5) and part of S6 for isofemale lines in this study (GenBank accessions: OL321811–OL321815). Exons in uppercase, with translated amino acids sequence overwritten. Codons wherein substitutions that lead to predicted amino acid changes are enclosed in boxes with responsible nucleotides highlighted. Positions of conserved amino acid changes at positions 918, 925, 929, 979 and 1014 among pyrethroid‐resistant aphids are indicated. Introns in lowercase, and cononical 5′‐gt and 3′‐ag intron/exon junctions underlined.

**Figure S2** Sanger sequence reads from the *A. glycines vgsc* gene encoding a portion of domain III segment 6 (DIII S6). Translated amino acid sequence is shown, and location of mutations M1524I, F1528L, F1538I, D1549V and E1553G associated with pyrethroid resistance in other insects are indicated accordingly. These mutations were not predicted in *A. glycines* isofemale lines. Heterozygote genotype present at co‐occurring nucleotide signals at three synonymous nucleotide positions are indicated by arrows.

**Figure S3** Multiple sequence alignment of *A. glycines vgsc* gene fragments encoding predicted α‐helical structures of domain III segment 6 (DII S6) from isofemale lines in this study (GenBank accessions: OL321816–OL321820). Three nucleic acid substitutions are predicted to be in 3rd codon positions and not cause amino acid changes (highlighted and encoded in boxes; heterozygote genotypes with co‐occurring A and G electropherogram peaks are indicated as an R, and co–co‐occurring T and G peaks as K; Fig. S2). Exons in uppercase, with translated amino acid sequences overwritten. Introns in lowercase, and cononical 5′‐gt and 3′‐ag intron/exon junctions underlined.

**Figure S4** Sanger sequence reads from the *A. glycines vgsc* gene, showing portion of the amplified domain IV segments 4 to 6 (DIV S4–S6) primers. This portion contains the single synonymous guanine (G) to adenosine (A) nucleotide substitution in the DIV S4‐S6 fragment indicated with an arrow, which is located in a leucine (L) 3rd codon position of the translated amino acid sequence.

**Figure S5** Multiple sequence alignment of *A. glycines vgsc* gene fragments encoding predicted α‐helical structures of domain IV segments 5 (DII S5) and D6 for isofemale lines in this study (GenBank accessions: OL321821–OL321825). The single predicted mutation is an adenosine (A) to guanine (G) transition in a leucine (L) 3rd codon position that is synonymous (nonamino acid changing), and heterozygote genotypes showing co‐occurring A and G electropherogram peaks (**Fig. S4**) are indicated as an R. Exons in uppercase, with translated amino acids sequence. Introns in lowercase, and cononical 5′‐gt and 3′‐ag intron/exon junctions underlined.

**Figure S6** (A) Kaplan–Meier survival analysis showing survival probability of *A. glycines* isofemale lines. (B) *P‐*values of pairwise comparisons of the survival probability of *A. glycines* isofemale lines using Log‐Rank test.

## Data Availability

The data that support the findings of this study are available from the corresponding author upon reasonable request.
