# Supplementary material for: Evidence of enhanced reproductive performance and lack‐of‐fitness costs among soybean aphids, Aphis glycines, with varying levels of pyrethroid resistance
Source: Pest Manag Sci. 2022 Mar 3;78(5):2000–10. doi: 10.1002/ps.6820 (PMC9310592; doi:10.1002/ps.6820)
Supplement: Supplementary file 1 — Figure S1 Multiple sequence alignment of A. glycines vgsc gene fragments encoding predicted α‐helical structures of domain II segment 5 (DII S5) and part of S6 for isofemale lines in this study (GenBank accessions: OL321811–OL321815). Exons in uppercase, with translated amino acids sequence overwritten. Codons wherein substitutions that lead to predicted amino acid changes are enclosed in boxes with responsible nucleotides highlighted. Positions of conserved amino acid changes at positions 918, 925, 929, 979 and 1014 among pyrethroid‐resistant aphids are indicated. Introns in lowercase, and cononical 5′‐gt and 3′‐ag intron/exon junctions underlined. [file PS-78-2000-s001.pdf]

Figure S1: Multiple sequence alignment of *Aphis glycines* voltage gated sodium channel (*vgsc*) gene fragments encoding predicted α-helical structures of domain II segment 5 (DII S5) and part of S6 for isofemale lines in this study (GenBank accessions: OL321811 – OL321815). Exons are in uppercase, with translated amino acids sequence overwritten. Codons wherein substitutions that lead to predicted amino acid changes are enclosed in boxes with responsible nucleotides highlighted. Positions of conserved amino acid changes at positions 918, 925, 929, 979, and 1014 among pyrethroid resistant aphids are indicated. Introns in lowercase, and cononical 5'-gt and 3'-ag intron/exon junctions underlined.

```
Sample        ----------intron----------[ L  R  V  F  K  L  A  K  S  W  P  T  L  N  L  L  I  S 916 Accession
Boone-2018    ctaatgatttgaaatgtttaactatagCTTCGAGTATTTAAGTTGGCAAAATCTTGGCCCACACTTAATCTTTTAATATC     OL321811
Nashua-2018   ctaatgatttgaaatgtttaactatagCTTCGAGTATTTAAGTTGGCAAAATCTTGGCCCACACTTAATCTTTTAATATC     OL321812
MN1_2017      ctaatgatttgaaatgtttaactatagCTTCGAGTATTTAAGTTGGCAAAATCTTGGCCCACACTTAATCTTTTAATATC     OL321813
Kanawha-2019  ctaatgatttgaaatgtttaactatagCTTCGAGTATTTAAGTTGGCAAAATCTTGGCCCACACTTAATCTTTTAATATC     OL321814
Darwin-2019   ctaatgatttgaaatgtttaactatagCTTCGAGTATTTAAGTTGGCAAAATCTTGGCCCACACTTAATCTTTTAATATC     OL321815
            1          .         .         .         .         :         .         .         .  80

                M918L/I              L925M        T929I
                                               ......................DII S5..................>
Sample          I  M  G  R  T  I  G  A  L  G  N  L  T  F  V  L  C  I  I  I  F  I  F  A  V  M   942 Accession
Boone-2018    AATAATGGGTCGAACCATTGGTGCTTTGGGTAACCTAACGTTTGTGTTGTGCATAATCATATTTATATTCGCCGTTATGG     OL321811
Nashua-2018   AATAATGGGTCGAACCATTGGTGCTTTGGGTAACCTAACGTTTGTGTTGTGCATAATCATATTTATATTCGCCGTTATGG     OL321812
MN1_2017      AATAATRGGTCGAACCATTGGTGCTTTGGGTAACCTAACGTTTGTGTTGTGCATAATCATATTTATATTCGCCGTTATGG     OL321813
Kanawha-2019  AATAATGGGTCGAACCATTGGTGCTTTGGGTAACCTAACGTTTGTGTTGTGCATAATCATATTTATATTCGCCGTTATGG     OL321814
Darwin-2019   AATAWTGGGTCGAACCATTGGTGCTWTGGGTAACCTAACGTTTGTGTTGTGCATAATCATATTTATATTCGCCGTTATGG     OL321815
           81          .         1         .         .         .         .         :         . 160

              .....DII S5......
Sample        G  M  Q  L  F  G  K  N  Y  T  ]--------------------------intron-----------------     Accession
Boone-2018    GTATGCAGTTATTTGGAAAAAACTACACAGgtaatctataatgttatcaaacaaacaaaatacgacggattacaaataaa     OL321811
Nashua-2018   GTATGCAGTTATTTGGAAAAAACTACACAGgtaatctataatgttatcaaacaaacaaaatacgacggattacaaataaa     OL321812
MN1_2017      GTATGCAGTTATTTGGAAAAAACTACACAGgtaatctataatgttatcaaacaaacaaaatacgacggattacaaataaa     OL321813
Kanawha-2019  GTATGCAGTTATTTGGAAAAAACTACACAGgtaatctataatgttatcaaacaaacaaaatacgacggattacaaataaa     OL321814
Darwin-2019   GTATGCAGTTATTTGGAAAAAACTACACAGgtaatctataatgttatcaaacaaacaaaatacgacggattacaaataaa     OL321815
          161          .         .         .         2         .         .         .         . 240

Sample        -----intron------[E  K  M  Y  L  F  K  D  H  E  L  P  R  W  N  F  T  D  F  L  H  973
Boone-2018    attgtgtttttcacagAAAAAATGTACTTATTCAAAGACCACGAGCTTCCCCGGTGGAACTTCACCGATTTTTTGCACT     OL321811
Nashua-2018   attgtgtttttcacagAAAAAATGTACTTATTCAAAGACCACGAGCTTCCCCGGTGGAACTTCACCGATTTTTTGCACT     OL321812
MN1_2017      attgtgtttttcacagAAAAAATGTACTTATTCAAAGACCACGAGCTTCCCCGGTGGAACTTCACCGATTTTTTGCACT     OL321813
Kanawha-2019  attgtgtttttcacagAAAAAATGTACTTATTCAAAGACCACGAGCTTCCCCGGTGGAACTTCACCGATTTTTTGCACT     OL321814
Darwin-2019   attgtgtttttcacagAAAAAATGTACTTATTCAAAGACCACGAGCTTCCCCGGTGGAACTTCACCGATTTTTTGCACT     OL321815
          241          :         .         .         .         .         3         .         . 320
```

```
                  F979S
Sample        S  F  M  I  V  F  R  V  L  C  G  E  W  I  E  S  M  W  D  C  L  H  V  G  E  P  T 1000 Accession
Boone-2018    CGTTTATGATAGTATTTCGAGTATTATGTGGTGAATGGATTGAATCAATGTGGGACTGCTTACACGTTGGAGAACCAACG     OL321811
Nashua-2018   CGTTTATGATAGTATTTCGAGTATTATGTGGTGAATGGATTGAATCAATGTGGGACTGCTTACACGTTGGAGAACCAACG     OL321812
MN1_2017      CGTTTATGATAGTATTTCGAGTATTATGTGGTGAATGGATTGAATCAATGTGGGACTGCTTACACGTTGGAGAACCAACG     OL321813
Kanawha-2019  CGTTTATGATAGTATTTCGAGTATTATGTGGTGAATGGATTGAATCAATGTGGGACTGCTTACACGTTGGAGAACCAACG     OL321814
Darwin-2019   CGTTTATGATAGTATTTCGAGTATTATGTGGTGAATGGATTGAATCAATGTGGGACTGCTTACACGTTGGAGAACCAACG     OL321815
          321          .         .         :         .         .         .         .         4  400
                                                L1014F
              <.................DII S6..................>
Sample         C  I  P  F  F  L  A  T  V  V  I  G  N  L  V ]---------------intron-------------     Accession
Boone-2018    TGTATACCATTCTTCTTGGCTACTGTTGTCATCGGTAACCTTGTGGgtatgtataagtactgacaatgcataaatgtgtat     OL321811
Nashua-2018   TGTATACCATTCTTCTTGGCTACTGTTGTCATCGGTAACYTTGTGGgtatgtataagtactgacaatgcataaatgtgtat     OL321812
MN1_2017      TGTATACCATTCTTCTTGGCTACTGTTGTCATCGGTAACYTTGTGGgtatgtataagtactgacaatgcataaatgtgtat     OL321813
Kanawha-2019  TGTATACCATTCTTCTTGGCTACTGTTGTCATCGGTAACTTTGTGGgtatgtataagtactgacaatgcataaatgtgtat     OL321814
Darwin-2019   TGTATACCATTCTTCTTGGCTACTGTTGTCATCGGTAACCTTGTGGgtatgtataagtactgacaatgcataaatgtgtat     OL321815
          401          .         .         .         .         :         .         .         .  480

Sample        -------------------------------------intron-------------------------------------     Accession
Boone-2018    tacttagggaaacatatttaataagatgcaaacggcgaggtggatgacaatttttaggattttaaatgtggcataatat     OL321811
Nashua-2018   tacttagggaaacatatttaataagatgcaaacggcgaggtggatgacaatttttaggattttaaatgtggcataatat     OL321812
MN1_2017      tacttagggaaacatatttaataagatgcaaacggcgaggtggatgacaatttttaggattttaaatgtggcataatat     OL321813
Kanawha-2019  tacttagggaaacatatttaataagatgcaaacggcgaggtggatgacaatttttaggattttaaatgtggcataatat     OL321814
Darwin-2019   tacttagggaaacatatttaataagatgcaaacggcgaggtggatgacaatttttaggattttaaatgtggcataatat     OL321815
          481         .         5         .         .         .         .         :         .   560

Sample        -------------------------------------intron-------------------------------------     Accession
Boone-2018    tactctccagaatttacaaccatcatccattatcattcagcaataaagtttatgtctcttacataaacatttttatcaca     OL321811
Nashua-2018   tactctccagaatttacaaccatcatccattatcattcggcaataaagtttatgtctcttacataaacatttttatcaca     OL321812
MN1_2017      tactctccagaatttacaaccatcatccattatcattcrgcaataaagtttatgtctcttacataaacatttttatcaca     OL321813
Kanawha-2019  tactctccagaatttacaaccatcatccattatcattcggcaataaagtttatgtctcttacataaacatttttatcaca     OL321814
Darwin-2019   tactctccagaatttacaaccatcatccattatcattcagcaataaagtttatgtctcttacataaacatttttatcaca
          561          .         .         .         6         .         .         .         .  640

Sample        -------------------------------------intron-------------------------------------     Accession
Boone-2018    tttaaaagccaaaataatttttaattcatatacaaatttaataaattgtatttgtaagataatctatggaatctcataat     OL321811
Nashua-2018   tttaaaagccaaaataatttttaattcatatacaaatttaataaattgtatttgtaagataatctatggaatctcataat     OL321812
MN1_2017      tttaaaagccaaaataatttttaattcatatacaaatttaataaattgtatttgtaagataatctatggaatctcataat     OL321813
Kanawha-2019  tttaaaagccaaaataatttttaattcatatacaaatttaataaattgtatttgtaagataatctatggaatctcataat     OL321814
Darwin-2019   tttaaaagccaaaataatttttaattcatatacaaatttaataaattgtatttgtaagataatctatggaatctcataat     OL321815
          641          :         .         .         .         .         7         .         .  720
```

```
Sample        --------------------------------------intron--------------------------------------     Accession
Boone-2018    ttaattttgttattatgccaaataaattattataatattgatgttatatttatatataactagtataaataactatagt     OL321811
Nashua-2018   ttaattttgttattatgccaaataaattactataatattgatgttatatttatatataactagtataaataactatagt     OL321812
MN1_2017      ttaattttgttattatgccaaataaattaytataatattgatgttatatttatatataactagtataaataactatagt     OL321813
Kanawha-2019  ttaattttgttattatgccaaataaattactataatattgatgttatatttatatataactagtataaataactatagt     OL321814
Darwin-2019   ttaattttgttattatgccaaataaattattataatattgatgttatatttatatataactagtataaataactatagt     OL321815
         721           .         .         :         .         .         .         .        8  800

Sample        --------------------------------------intron--------------------------------------     Accession
Boone-2018    tacaatttagtgatactatacatgtattataatatgtatagaaaatctttaatgtagttaactactttaacgtttaaaaa     OL321811
Nashua-2018   tacaatttagtgatactatacatgtattataatatgtatagaaaatctttaatgtagttaactactttaacgtttaaaaa     OL321812
MN1_2017      tacaatttagtgatactatacatgtattataatatgtatagaaaatctttaatgtagttaactactttaacgtttaaaaa     OL321813
Kanawha-2019  tacaatttagtgatactatacatgtattataatatgtatagaaaatctttaatgtagttaactactttaacgtttaaaaa     OL321814
Darwin-2019   tacaatttagtgatactatacatgtattataatatgtatagaaaatctttaatgtagttaactactttaacgtttaaaaa     OL321815
         801           .         .         .         .         :         .         .         .  880

Sample        --------------------------------------intron--------------------------------------     Accession
Boone-2018    acaaatcagcataattatttaatataatactaattatcgcttagttattttaaatttcaatcatatactttattaatata     OL321811
Nashua-2018   acaaatcagcataattatttaatataatactaattatcgcttagttattttaaatttcaatcatatactttattaatata     OL321812
MN1_2017      acaaatcagcataattatttaatataatactaattatcgcttagttattttaaatttcaatcatatactttattaatata     OL321813
Kanawha-2019  acaaatcagcataattatttaatataatactaattatcgcttagttattttaaatttcaatcatatactttattaatata     OL321814
Darwin-2019   acaaatcagcataattatttaatataatactaattatcgcttagttattttaaatttcaatcatatactttattaatata     OL321815
         881           .         9         .         .         .         .         :         .  960

Sample        --------------------------------------intron--------------------------------------     Accession
Boone-2018    gccatttatttttgtatttgttattttcattactttttttacttgattttgataattttctgaaaacattagcatattaca     OL321811
Nashua-2018   gccatttatttttgtatttgttattttcattactttttttacttgattttgataattttctgaaaacattagcatattaca     OL321812
MN1_2017      gccatttatttttgtatttgttattttcattactttttttacttgattttgataattttctgaaaacattagcatattaca     OL321813
Kanawha-2019  gccatttatttttgtatttgttattttcattactttttttacttgattttgataattttctgaaaacattagcatattaca     OL321814
Darwin-2019   gccatttatttttgtatttgttattttcattactttttttacttgattttgataattttctgaaaacattagcatattaca     OL321815
         961           .         .         .         0         .         .         .         . 1040

Sample        --------------------------------------intron--------------------------------------     Accession
Boone-2018    atgactagtaaaataaaaaaaaaactatgtggcgcagattctaaaaatttagccataaaacaaattgaattattcacttg     OL321811
Nashua-2018   atgactagtaaaataaaaaaaaaactatgtggcgcagattctaaaaatttagccataaaacaaattgaattattcacttg     OL321812
MN1_2017      atgactagtaaaataaaaaaaaaactatgtggcgcagattctaaaaatttagccataaaacaaattgaattattcacttg     OL321813
Kanawha-2019  atgactagtaaaataaaaaaaaaactatgtggcgcagattctaaaaatttagccataaaacaaattgaattattcacttg     OL321814
Darwin-2019   atgactagtaaaataaaaaaaaaactatgtggcgcagattctaaaaatttagccataaaacaaattgaattattcacttg     OL321815
        1041           :         .         .         .         .         1         .         . 1120
```

```
Sample        -------------------------intron----------------------------------     Accession
Boone-2018    ttgtttctataaaattaataaattaaataattattttatgatatagccttgaagccacatgtatg     OL321811
Nashua-2018   ttgtttctataaaattaataaattaaataattattttatgatatagccttgaagccacatgtatg     OL321812
MN1_2017      ttgtttctataaaattaataaattaaataattattttatgatatagccttgaagccacatgtatg     OL321813
Kanawha-2019  ttgtttctataaaattaataaattaaataattattttatgatatagccttgaagccacatgtatg     OL321814
Darwin-2019   ttgtttctataaaattaataaattaaataattattttatgatatagccttgaagccacatgtatg     OL321815
        1121           .         .         :         .         .         .   1185
```
