# Supplementary material for: Evidence of enhanced reproductive performance and lack‐of‐fitness costs among soybean aphids, Aphis glycines, with varying levels of pyrethroid resistance
Source: Pest Manag Sci. 2022 Mar 3;78(5):2000–10. doi: 10.1002/ps.6820 (PMC9310592; doi:10.1002/ps.6820)

Figure S2: Sanger sequence reads from the *Aphis glycines* voltage-gated sodium channel (*vgsc*) gene encoding a portion of domain III segment 6 (DIII S6). Translated amino acid sequence is shown, and location of mutations M1524I, F1528L, F1538I, D1549V and E1553G associated with pyrethroid resistance in other insects are indicated accordingly. These mutations were not predicted in *A. glycines* isofemale lines. Heterozygote genotype present at co-occurring nucleotide signals in at three synonymous nucleotide positions are indicated by arrows.

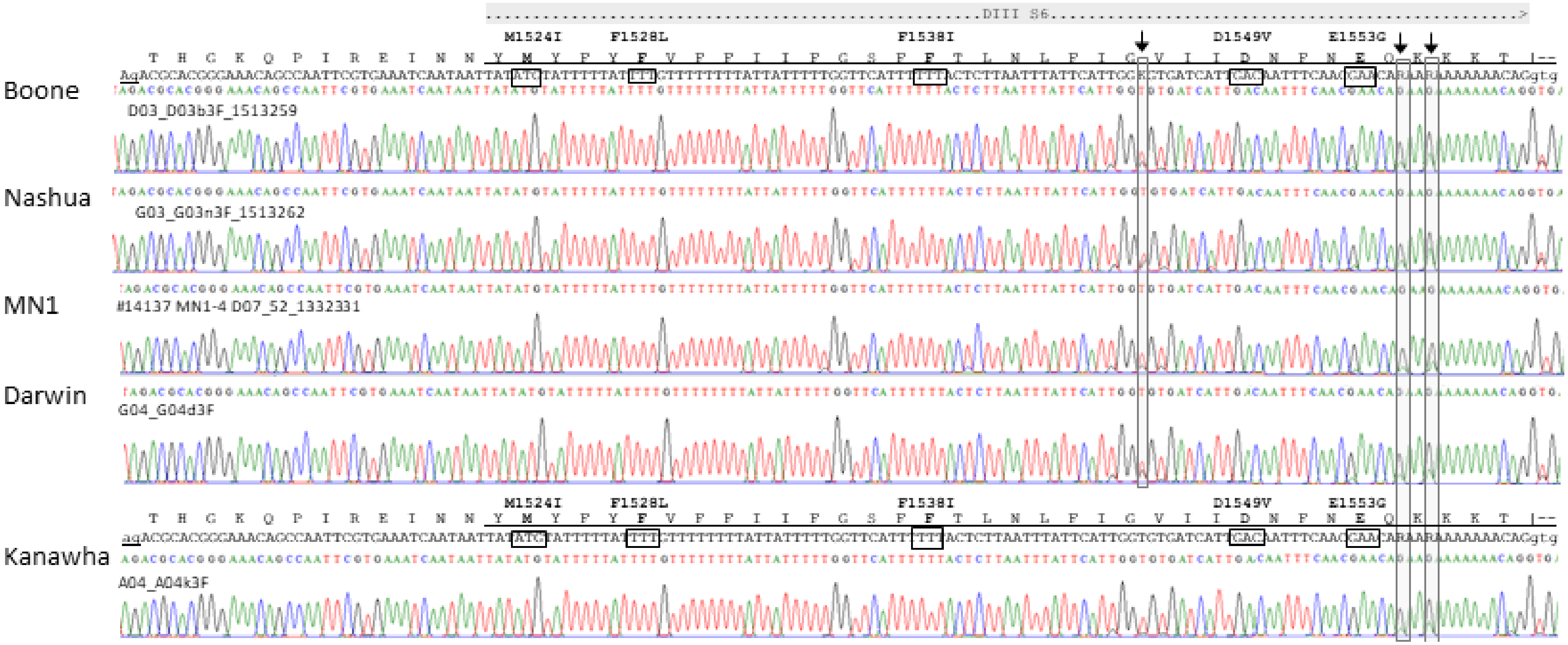

Supplement: Supplementary file 2 — Figure S2 Sanger sequence reads from the A. glycines vgsc gene encoding a portion of domain III segment 6 (DIII S6). Translated amino acid sequence is shown, and location of mutations M1524I, F1528L, F1538I, D1549V and E1553G associated with pyrethroid resistance in other insects are indicated accordingly. These mutations were not predicted in A. glycines isofemale lines. Heterozygote genotype present at co‐occurring nucleotide signals at three synonymous nucleotide positions are indicated by arrows. [file PS-78-2000-s002.pdf]
