# Supplementary material for: Evidence of enhanced reproductive performance and lack‐of‐fitness costs among soybean aphids, Aphis glycines, with varying levels of pyrethroid resistance
Source: Pest Manag Sci. 2022 Mar 3;78(5):2000–10. doi: 10.1002/ps.6820 (PMC9310592; doi:10.1002/ps.6820)
Supplement: Supplementary file 3 — Figure S3 Multiple sequence alignment of A. glycines vgsc gene fragments encoding predicted α‐helical structures of domain III segment 6 (DII S6) from isofemale lines in this study (GenBank accessions: OL321816–OL321820). Three nucleic acid substitutions are predicted to be in 3rd codon positions and not cause amino acid changes (highlighted and encoded in boxes; heterozygote genotypes with co‐occurring A and G electropherogram peaks are indicated as an R, and co–co‐occurring T and G peaks as K; Fig. S2). Exons in uppercase, with translated amino acid sequences overwritten. Introns in lowercase, and cononical 5′‐gt and 3′‐ag intron/exon junctions underlined. [file PS-78-2000-s004.pdf]

Figure S3: Multiple sequence alignment of *Aphis glycines* voltage gated sodium channel (*vgsc*) gene fragments encoding predicted α-helical structures of domain III segment 6 (DII S6) from isofemale lines in this study (GenBank accessions: OL321816 – OL321820). Three predicted nucleic acid substitutions are predicted to be in 3rd codon positions and not cause amino acid changes (highlighted and encoded in boxes; heterozygote genotypes with co-occurring A and G electropherogram peaks are indicated as an R, and co-co-occurring T and G peaks as K; Figure S2). All substitutions, where all Exons are in uppercase, with translated amino acids sequence overwritten. Introns in lowercase, and cononical 5'-gt and 3'-ag intron/exon junctions underlined.

```
Sample        <---------------------------------------- Intron -------------------------------- Accession
Boone-2018    taaaaaaataaaaatatattatataagtatacctataaatgtctttttttgacatacatttataaaatactgatgtatat OL321816
Nashua-2018   taaaaaaataaaaatatattatataagtatacctataaatgtctttttttgacatacatttataaaatactgatgtatat OL321817
MN1_2017      taaaaaaataaaaatatattatataagtatacctataaatgtctttttttgacatacatttataaaatactgatgtatat OL321818
Kanawha-2019  taaaaaaataaaaatatattatataagtatacctataaatgtctttttttgacatacatttataaaatactgatgtatat OL321819
Darwin-2019   taaaaaaataaaaatatattatataagtatacctataaatgtctttttttgacatacatttataaaatactgatgtatat OL321820
            1          .         .         .         .         :         .         .         .   80

                                                                      ..............DIII S6............
Sample        ----------[ T  H  G  K  Q  P  I  R  E  I  N  N  Y  M  Y  F  Y  F  V  F  F  I  I  Accession
Boone-2018    ttgttattaagACGCACGGGAAACAGCCAATTCGTGAAATCAATAATTATATGTATTTTTATTTTGTTTTTTTTATTATT OL321816
Nashua-2018   ttgttattaagACGCACGGGAAACAGCCAATTCGTGAAATCAATAATTATATGTATTTTTATTTTGTTTTTTTTATTATT OL321817
MN1_2017      ttgttattaagACGCACGGGAAACAGCCAATTCGTGAAATCAATAATTATATGTATTTTTATTTTGTTTTTTTTATTATT OL321818
Kanawha-2019  ttgttattaagACGCACGGGAAACAGCCAATTCGTGAAATCAATAATTATATGTATTTTTATTTTGTTTTTTTTATTATT OL321819
Darwin-2019   ttgttattaagACGCACGGGAAACAGCCAATTCGTGAAATCAATAATTATATGTATTTTTATTTTGTTTTTTTTATTATT OL321820
           81          .         1         .         .         .         .         :         .  160

              .................DIII S6....................
Sample         F  G  S  F  F  T  L  N  L  F  I  G  V  I  I  D  N  F  N  E  Q  K  K  K  T  ]--- Accession
Boone-2018    TTTGGTTCATTTTTTACTCTTAATTTATTCATTGGKGTGATCATTGACAATTTCAACGAACARAARAAAAAAACAGgtga OL321816
Nashua-2018   TTTGGTTCATTTTTTACTCTTAATTTATTCATTGGKGTGATCATTGACAATTTCAACGAACARAARAAAAAAACAGgtga OL321817
MN1_2017      TTTGGTTCATTTTTTACTCTTAATTTATTCATTGGKGTGATCATTGACAATTTCAACGAACARAARAAAAAAACAGgtga OL321818
Kanawha-2019  TTTGGTTCATTTTTTACTCTTAATTTATTCATTGGTGTGATCATTGACAATTTCAACGAACARAARAAAAAAACAGgtga OL321819
Darwin-2019   TTTGGTTCATTTTTTACTCTTAATTTATTCATTGGKGTGATCATTGACAATTTCAACGAACARAARAAAAAAACAGgtga OL321820
          161          .         .         .         2         .         .         .         .  240

Sample        ----------------------------------------- Intron -------------------------------- Accession
Boone-2018    cttattttaaacatatagaatcaattagtattaattatttttttttttaaggtactcaagaaatatcgatataagttcata OL321816
Nashua-2018   cttattttaaacatatagaatcaattagtattaattatttttttttttaaggtactcaagaaatatcgatataagttcata OL321817
MN1_2017      cttattttaaacatatagaatcaattagtattaattatttttttttttaaggtactcaagaaatatcgatataagttcata OL321818
Kanawha-2019  cttattttaaacatatagaatcaattagtattaattatttttttttttaaggtactcaagaaatatcgatataagttcata OL321819
Darwin-2019   cttattttaaacatatagaatcaattagtattaattatttttttttttaaggtactcaagaaatatcgatataagttcata OL321820
          241          :         .         .         .         .         3         .         .  320
```

```
Sample       ------------ Intron ------------> Accession
Boone-2018   actatattatgggttaccattttaaaaacaata OL321816
Nashua-2018  actatattatgggttaccattttaaaaacaata OL321817
MN1_2017     actatattatgggttaccattttaaaaacaata OL321818
Kanawha-2019 actatattatgggttaccattttaaaaacaata OL321819
Darwin-2019  actatattatgggttaccattttaaaaacaata OL321820
         321          .         .         :     353
```
