# Supplementary material for: Evidence of enhanced reproductive performance and lack‐of‐fitness costs among soybean aphids, Aphis glycines, with varying levels of pyrethroid resistance
Source: Pest Manag Sci. 2022 Mar 3;78(5):2000–10. doi: 10.1002/ps.6820 (PMC9310592; doi:10.1002/ps.6820)

Figure S4: Sanger sequence reads from the *Aphis glycines* voltage-gated sodium channel (*vgsc*) gene, showing portion of the amplified domain IV segments 4 to 6 (DIV S4-S6) primers. This portion contains the single synonymous guanine (G) to adenosine (A) nucleotide substitution in the DIV S4-S6 fragment indicated with an arrow, which is located in a leucine (L) 3rd codon position of the translated amino acid sequence.

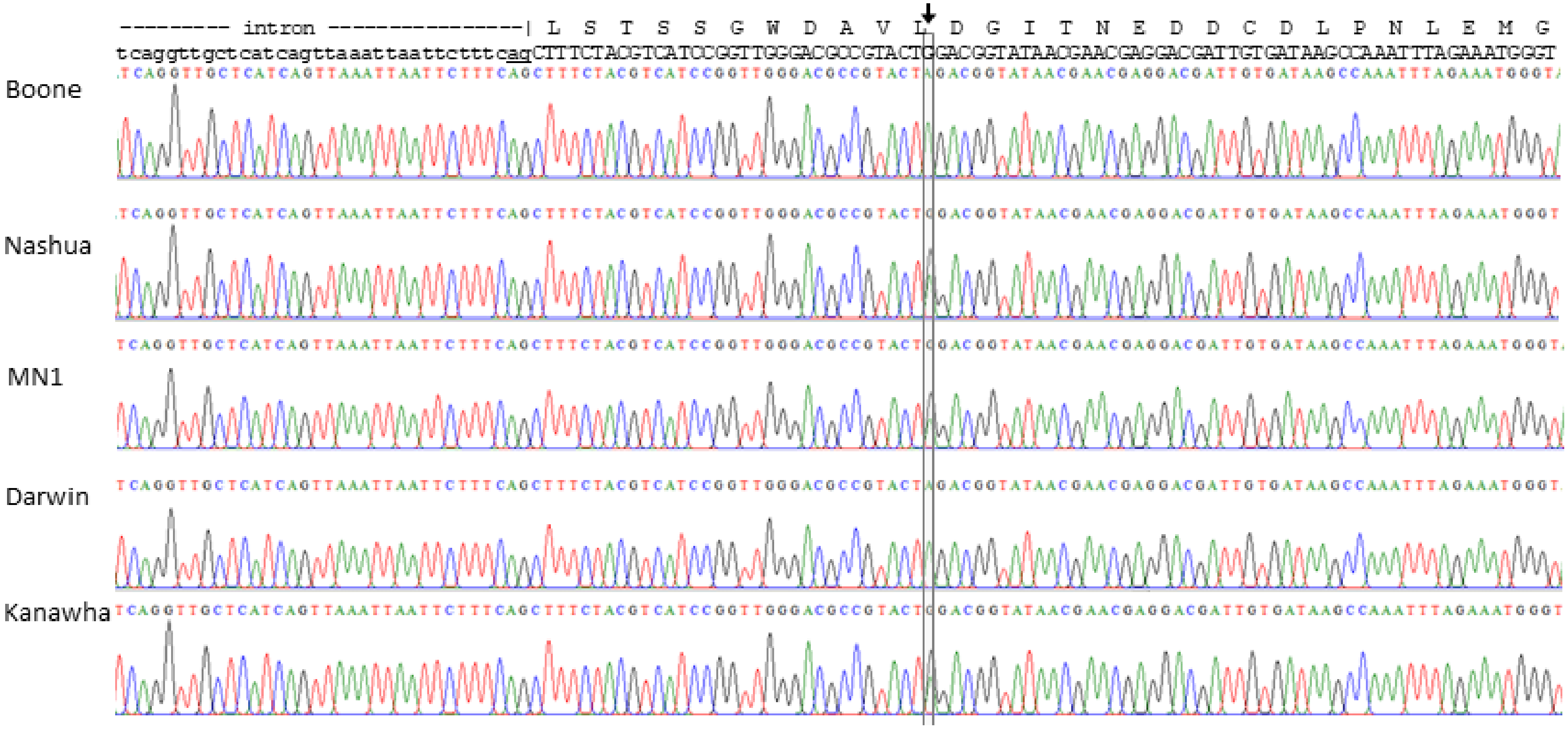

Supplement: Supplementary file 4 — Figure S4 Sanger sequence reads from the A. glycines vgsc gene, showing portion of the amplified domain IV segments 4 to 6 (DIV S4–S6) primers. This portion contains the single synonymous guanine (G) to adenosine (A) nucleotide substitution in the DIV S4‐S6 fragment indicated with an arrow, which is located in a leucine (L) 3rd codon position of the translated amino acid sequence. [file PS-78-2000-s003.pdf]
