# Supplementary material for: Evidence of enhanced reproductive performance and lack‐of‐fitness costs among soybean aphids, Aphis glycines, with varying levels of pyrethroid resistance
Source: Pest Manag Sci. 2022 Mar 3;78(5):2000–10. doi: 10.1002/ps.6820 (PMC9310592; doi:10.1002/ps.6820)
Supplement: Supplementary file 5 — Figure S5 Multiple sequence alignment of A. glycines vgsc gene fragments encoding predicted α‐helical structures of domain IV segments 5 (DII S5) and D6 for isofemale lines in this study (GenBank accessions: OL321821–OL321825). The single predicted mutation is an adenosine (A) to guanine (G) transition in a leucine (L) 3rd codon position that is synonymous (nonamino acid changing), and heterozygote genotypes showing co‐occurring A and G electropherogram peaks (Fig. S4) are indicated as an R. Exons in uppercase, with translated amino acids sequence. Introns in lowercase, and cononical 5′‐gt and 3′‐ag intron/exon junctions underlined. [file PS-78-2000-s006.pdf]

**Figure S5:** Multiple sequence alignment of *Aphis glycines* voltage gated sodium channel (*vgsc*) gene fragments encoding predicted α-helical structures of domain IV segments 5 (DII S5) and D6 for isofemale lines in this study (GenBank accessions: OL321821 – OL321825). The single predicted mutation is an adenosine (A) to guanine (G) transition in a leucine (L) 3rd codon position that is synonymous (non-amino acid changing), and heterozygote genotypes showing co-occurring A and G electropherogram peaks (**Supplementary Figure S4**) are indicated as an R. Exons are in uppercase, with translated amino acids sequence. Introns are in lowercase, and cononical 5'-gt and 3'-ag intron/exon junctions underlined.

```
              ......DIV S4........                                                  ......DIV S5.....>
Sample           A  K  G  I  R  T  L  L  F  A  L  A  M  S  L  P  A  L  F  N  I  C  L  L  L  F  Accession
Boone-2018    GAGCGAAAGGGATTAGAACTCTATTGTTCGCTTTAGCCATGTCACTTCCAGCGTTATTCAACATATGTTTGTTACTGTTT OL321821
Nashua-2018   GAGCGAAAGGGATTAGAACTCTATTGTTCGCTTTAGCCATGTCACTTCCAGCGTTATTCAACATATGTTTGTTACTGTTT OL321822
MN1_2017      GAGCGAAAGGGATTAGAACTCTATTGTTCGCTTTAGCCATGTCACTTCCAGCGTTATTCAACATATGTTTGTTACTGTTT OL321823
Kanawha-2019  GAGCGAAAGGGATTAGAACTCTATTGTTCGCTTTAGCCATGTCACTTCCAGCGTTATTCAACATATGTTTGTTACTGTTT OL321824
Darwin-2019   GAGCGAAAGGGATTAGAACTCTATTGTTCGCTTTAGCCATGTCACTTCCAGCGTTATTCAACATATGTTTGTTACTGTTT OL321825
           1          .         .         .         .         :         .         .         .    80

              <..................DIV S5.........................
Sample         L  V  M  F  I  F  A  I  F  G  M  S  F  F  M  N  V  D  N  H  G  G  L  D  E  D  Y Accession
Boone-2018    CTTGTCATGTTCATATTTGCTATATTTGGCATGTCGTTTTTCATGAACGTAGATAATCATGGAGGTTTGGATGAGGATTA OL321821
Nashua-2018   CTTGTCATGTTCATATTTGCTATATTTGGCATGTCGTTTTTCATGAACGTAGATAATCATGGAGGTTTGGATGAGGATTA OL321822
MN1_2017      CTTGTCATGTTCATATTTGCTATATTTGGCATGTCGTTTTTCATGAACGTAGATAATCATGGAGGTTTGGATGAGGATTA OL321823
Kanawha-2019  CTTGTCATGTTCATATTTGCTATATTTGGCATGTCGTTTTTCATGAACGTAGATAATCATGGAGGTTTGGATGAGGATTA OL321824
Darwin-2019   CTTGTCATGTTCATATTTGCTATATTTGGCATGTCGTTTTTCATGAACGTAGATAATCATGGAGGTTTGGATGAGGATTA OL321825
          81          .         1         .         .         .         .         :         .   160

Sample          N  F  R  T  F  G  Q  S  M  I  L  L  F  M ]-------------- intron -------------- Accession
Boone-2018    CAATTTCCGCACTTTCGGGCAATCGATGATACTTCTTTTCATGgtaagcttataatcggtcctatcaatgtcacgcctga OL321821
Nashua-2018   CAATTTCCGCACTTTCGGGCAATCGATGATACTTCTTTTCATGgtaagcttataatcggtcctatcaatgtcacgcctga OL321822
MN1_2017      CAATTTCCGCACTTTCGGGCAATCGATGATACTTCTTTTCATGgtaagcttataatcggtcctatcaatgtcacgcctga OL321823
Kanawha-2019  CAATTTCCGCACTTTCGGGCAATCGATGATACTTCTTTTCATGgtaagcttataatcggtcctatcaatgtcacgcctga OL321824
Darwin-2019   CAATTTCCGCACTTTCGGGCAATCGATGATACTTCTTTTCATGgtaagcttataatcggtcctatcaatgtcacgcctga OL321825
         161          .         .         .         2         .         .         .         .   240

Sample        ---------------------------------- intron ------------------------------------- Accession
Boone-2018    tgaaaatagtgattctataatatttagtgtgccataataatatcacctagtctatcaggttgctcatcagttaaattaa OL321821
Nashua-2018   tgaaaatagtgattctataatatttagtgtgccataataatatcacctagtctatcaggttgctcatcagttaaattaa OL321822
MN1_2017      tgaaaatagtgattctataatatttagtgtgccataataatatcacctagtctatcaggttgctcatcagttaaattaa OL321823
Kanawha-2019  tgaaaatagtgattctataatatttagtgtgccataataatatcacctagtctatcaggttgctcatcagttaaattaa OL321824
Darwin-2019   tgaaaatagtgattctataatatttagtgtgccataataatatcacctagtctatcaggttgctcatcagttaaattaa OL321825
         241          :         .         .         .         .         3         .         .   320
```

```
Sample        - intron[ L  S  T  S  S  G  W  D  A  V  L  D  G  I  T  N  E  D  D  C  D  L  P  N Accession
Boone-2018    ttctttcagCTTTCTACGTCATCCGGTTGGGACGCCGTACTAGACGGTATAACGAACGAGGACGATTGTGATAAGCCAAA OL321821
Nashua-2018   ttctttcagCTTTCTACGTCATCCGGTTGGGACGCCGTACTRGACGGTATAACGAACGAGGACGATTGTGATAAGCCAAA OL321822
MN1_2017      ttctttcagCTTTCTACGTCATCCGGTTGGGACGCCGTACTRGACGGTATAACGAACGAGGACGATTGTGATAAGCCAAA OL321823
Kanawha-2019  ttctttcagCTTTCTACGTCATCCGGTTGGGACGCCGTACTRGACGGTATAACGAACGAGGACGATTGTGATAAGCCAAA OL321824
Darwin-2019   ttctttcagCTTTCTACGTCATCCGGTTGGGACGCCGTACTAGACGGTATAACGAACGAGGACGATTGTGATAAGCCAAA OL321825
          321          .         .         :         .         .         .         .        4  400

                                                ..................DIV S6...............
Sample          L  E  M  G  I  T  G  S  C  G  S  S  A  V  G  T  A  F  L  L  S  Y  L  V  I  N   Accession
Boone-2018    TTTAGAAATGGGTATCACGGGAAGTTGTGGAAGTAGTGCCGTGGGCACGGCATTTTTACTCTCGTACCTGGTGATCAATT OL321821
Nashua-2018   TTTAGAAATGGGTATCACGGGAAGTTGTGGAAGTAGTGCCGTGGGCACGGCATTTTTACTCTCGTACCTGGTGATCAATT OL321822
MN1_2017      TTTAGAAATGGGTATCACGGGAAGTTGTGGAAGTAGTGCCGTGGGCACGGCATTTTTACTCTCGTACCTGGTGATCAATT OL321823
Kanawha-2019  TTTAGAAATGGGTATCACGGGAAGTTGTGGAAGTAGTGCCGTGGGCACGGCATTTTTACTCTCGTACCTGGTGATCAATT OL321824
Darwin-2019   TTTAGAAATGGGTATCACGGGAAGTTGTGGAAGTAGTGCCGTGGGCACGGCATTTTTACTCTCGTACCTGGTGATCAATT OL321825
          401          .         .         .         .         :         .         .         .  480

              ..................DIV S6..............
Sample        F  L  I  V  I  N  M  Y  I  A  V  I  L  E  N  Y  S  Q  A  Accession
Boone-2018    TCCTCATCGTCATAAACATGTACATCGCAGTCATTCTGGAGAACTATTCACAGGCT OL321821
Nashua-2018   TCCTCATCGTCATAAACATGTACATCGCAGTCATTCTGGAGAACTATTCACAGGCT OL321822
MN1_2017      TCCTCATCGTCATAAACATGTACATCGCAGTCATTCTGGAGAACTATTCACAGGCT OL321823
Kanawha-2019  TCCTCATCGTCATAAACATGTACATCGCAGTCATTCTGGAGAACTATTCACAGGCT OL321824
Darwin-2019   TCCTCATCGTCATAAACATGTACATCGCAGTCATTCTGGAGAACTATTCACAGGCT OL321825
          481          .         5         .         .         .      536
```
