# Supplementary material for: Evidence of enhanced reproductive performance and lack‐of‐fitness costs among soybean aphids, Aphis glycines, with varying levels of pyrethroid resistance
Source: Pest Manag Sci. 2022 Mar 3;78(5):2000–10. doi: 10.1002/ps.6820 (PMC9310592; doi:10.1002/ps.6820)
Supplement: Supplementary file 6 — Figure S6 (A) Kaplan–Meier survival analysis showing survival probability of A. glycines isofemale lines. (B) P‐values of pairwise comparisons of the survival probability of A. glycines isofemale lines using Log‐Rank test. [file PS-78-2000-s005.pdf]

**Figure S6.** (A) Kaplan-Meier survival analysis showing survival probability of *Aphis glycines* isofemale lines. (B) *P-values* of pairwise comparisons of the survival probability of *A. glycines* isofemale lines using Log-Rank test.

**A)**

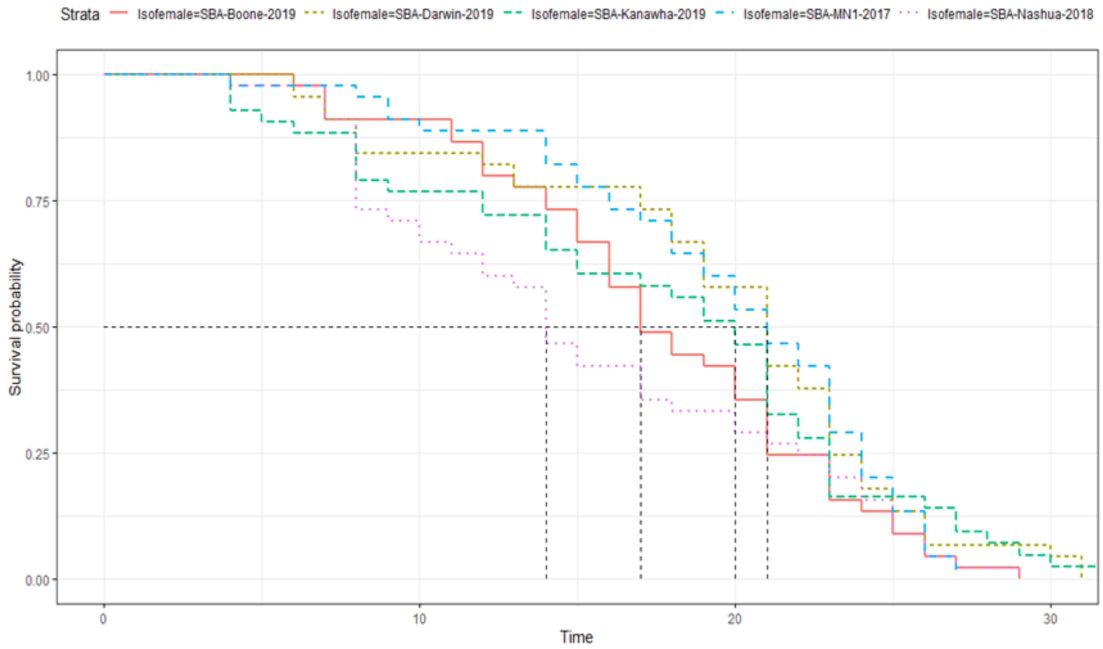


**B)**

| Isofemale line | SBA-Boone-2019 | SBA-Nashua-2018 | SBA-MN1-2017 | SBA-Kanawha-2019 | SBA-Darwin-2019 |
|---|---|---|---|---|---|
| SBA-Boone-2019 | - | 0.42 | 0.16 | 0.33 | 0.071 |
| SBA-Nashua-2018 | | - | 0.061 | 0.14 | 0.036 |
| SBA-MN1-2017 | | | - | 0.84 | 0.85 |
| SBA-Kanawha-2019 | | | | - | 0.53 |
| SBA-Darwin-2019 | | | | | - |
